# Estimation of the Continuous Pronation–Supination Movement by Using Multichannel EMG Signal Features and Kalman Filter: Application to Control an Exoskeleton

**DOI:** 10.3389/fbioe.2021.771255

**Published:** 2022-03-01

**Authors:** Lei Zhang, Jingang Long, RongGang Zhao, Haoyang Cao, Kai Zhang

**Affiliations:** School of Mechanical and Electrical Engineering, Xi’an Polytechnic University, Xi’an, China

**Keywords:** sEMG, DOF, exoskeleton robot, kalman filter, pronation–supination movement

## Abstract

The Hill muscle model can be used to estimate the human joint angles during continuous movement. However, adopting this model requires the knowledge of many parameters, such as the length and speed of contraction of muscle fibers, which are liable to change with different individuals, leading to errors in estimation. This study established the backpropagation neural network model based on surface electromyography (sEMG) features and human movement angle. First, the function of muscles in joint rotation is defined, and then, sensors are placed on muscle tissues to gain sEMG, and then, a relation model between the surface sEMG features and the joint angle is constructed. As integrated electromyography information cannot be well reflected through a single electromyography feature, a feature extraction method combining the time domain, frequency domain, and time–frequency domain was proposed. As the degree of freedom (DOF) of the pronation–supination movement was controlled by several muscles, it was difficult to make an angle prediction. A method of correcting the estimation error based on the Kalman filter was raised to cope with this problem. An exoskeleton robot with one DOF was designed and put into the tracking experiment. The results show that the proposed model was able to enhance the estimation of the joint angle during continuous pronation–supination movements.

## 1 Introduction

In the analysis of human motion, one of the most challenging issues is related to the possibility of estimating the joint kinematics during the execution of the continuous movement.

The current research (Han et al., 2015; Li et al., 2017) on the continuous movement estimation of limbs mainly focuses on using various surface electromyography (sEMG) features to estimate the joint angle, and there are two ways to achieve it. The first is to establish an articulation dynamics model with muscle physiology involved, which takes sEMG as the input and then calculates joint torque; the second is to set the regression relationship of sEMG and the articulation movement angle (Gijsberts et al., 2014; Hahne et al., 2014; Kuang et al., 2017; Long et al., 2017; Wang et al., 2017; Zhang et al., 2017).

The Hill model is one of the existing biomechanical models used frequently by researchers. In 1938, Hill used the frog’s sartorius muscle as an experimental sample and observed the relationship between the muscle contraction force and muscle contraction speed, known as the Hill muscle model. The model was simplified into three components: contraction, series elasticity, and parallel elasticity. This is the first model to have successfully described the changes of muscle contraction and is gradually developed as a normal way of predicting the joint angle (Huang et al., 2020; Xi et al., 2021). For example, Cavallaro et al. (2006) designed an upper limb exoskeleton robot system and used the improved Hill model to conduct continuous movement estimation, and they proposed a 28-channel signal acquisition instrument to obtain sEMG, realizing the continuous angle estimation of the upper limb. Pang et al. (Pang et al., 2013) established the Hill model of finger flexion and finger extension through acquired sEMG of the superficial flexor and extensor muscles, and then, they processed sEMG using the Kalman filter. The experiment was conducted on five subjects, suggesting that the Hill model they designed could complete the finger flexion angle estimation.

Diverse machine learning algorithms are utilized to establish the relationship between sEMG and the joint angle (Chen et al., 2021; Padhy 2021; Xue et al., 2021), whose process is easy and no complex calculations are involved. For example, Xiao et al. (Xiao et al., 2017) used the average absolute value, waveform length, zero-crossing points, and the number of slope sign changes to extract time-domain features, and they proposed a gray feature-weighted support vector machine to construct models of sEMG and elbow joint angles. Ding et al. (2017) divided sEMG into redundant and non-redundant sub-vectors, established a state-space motion model, and built a closed-loop correction algorithm to predict the upper limb elbow joint angle.

The elbow movement is mainly powered by biceps and triceps. The sEMG strength and quality gained from the biceps and triceps are better than that of the upper limb muscle. Existing research on the upper limb movement angle mainly focuses on the elbow joint. Gui et al. (Qizheng 2016) proposed an upper limb joint angle estimation method based on the support vector regression and muscle coordination model. The experimental results indicated that the elbow joint movement angle estimation showed higher accuracy. Raj et al. (Raj and Sivanandan 2017) placed EMG sensor electrodes on users’ biceps to acquire sEMG. Three different models were utilized to estimate the elbow joint’s angular displacement and velocity during continuous flexion and extension. The test results suggested that the adaptive neuro-fuzzy inference system showed the best accuracy among the three models. Sommer et al. (2018) used 3-channel EMG sensors to record biceps, triceps, and radial muscle sEMG and established an external input autoregressive model to predict the elbow continuous movement angle. Li et al. (2018) utilized 4-channel EMG sensors to obtain sEMG of subjects’ elbows. They used sEMG as the input and the joint angle as the output, and a continuous movement estimation model of the elbow joint was established. Xiao et al. (2018) obtained five time-domain features of sEMG from biceps and triceps, including the average absolute value, waveform length, zero-crossing time, number of changes in slope sign, and standard deviation. The random forest was utilized to predict the angle of the elbow joint. The results suggested that when the angular velocity of the joint rotation was 15–180.0°/s, more accuracy could be obtained.

Existing studies employed muscle physiology to establish a joint movement model with sEMG as its input. However, this model has its shortcomings: the difficulty in getting the knowledge of many physiological parameters and the complicated parameters. Thus, some studies sought to optimize parameters and achieved good results. For example, Ramos and Meggiolaro (2014) optimized the Hill parameters through a genetic algorithm and established an improved human elbow joint movement angle estimation model. Nevertheless, it introduced a new problem, namely, the selection and applicability of parameter optimization algorithms. Other research works (Xiao et al., 2017; Li et al., 2018) established the relationship model of the machine learning algorithm of sEMG and the joint angle, which focuses mainly on the upper and lower limbs, and apart from the elbow joint, other parts of upper limbs are less involved. The pronation–supination movement is controlled by multiple groups of muscles, so it is difficult to estimate the action by using an established relationship model; hence research on them is scarce.

In this study, the movement intentions of the upper limbs of one degree of freedom (DOF) were identified and tracked continuously by the exoskeleton robot. First, the study analyzed the preprocessing method of sEMG. We extracted features from the time domain, frequency domain, and time–frequency domain to obtain comprehensive signal information. After analyzing the structure of the upper limb, we designed an exoskeleton robot with one DOF. The backpropagation (BP) neural network model of sEMG features and the joint angle was constructed based on their relationship. As the DOF of the pronation–supination movement was controlled by several muscles, it was difficult to make an angle prediction. Hence, a method of correcting the estimation error based on the Kalman filter was raised. One DOF tracking test was performed to verify to what extent the established BP neural network model is effective in the recognizing movement intention.

## 2 Methods and Materials

### 2.1 Surface Electromyography Acquisition

**FIGURE 1:**
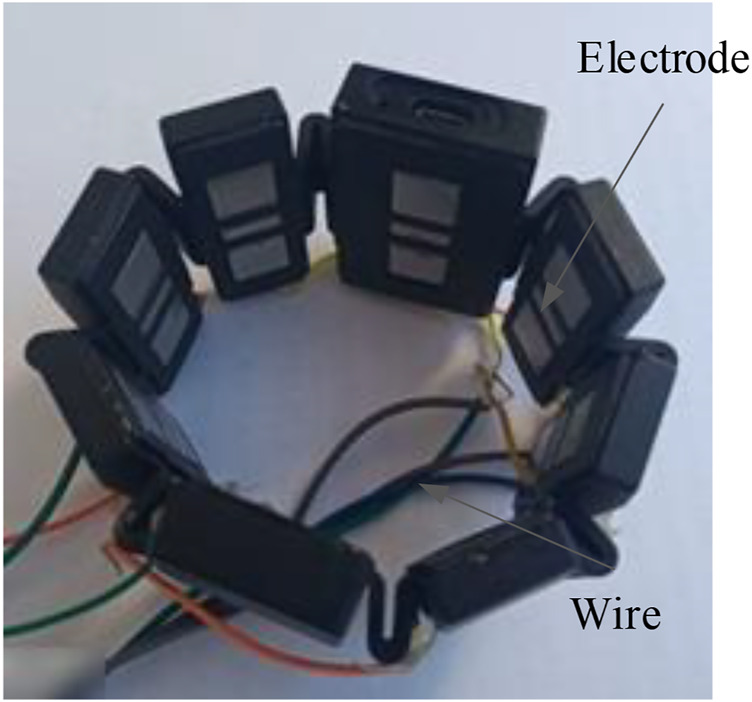
Circumferential electromyography equipment.

The 8-channel myoelectric ring (Dting-One) produced by Beech Innovation Company was employed to obtain sEMG, as shown in Figure 1. The signals received from every channel were amplified 700 times and then transmitted by Bluetooth to the computer. In order to improve the acquisition phase, the Bluetooth channel was bypassed by directly drawing five signals through wired connections so as to increase the sampling frequency from 100 to 1,000 Hz; the five signals were directly associated with five of the main forearm muscles, as hereinafter reported. The signals’ processing and analysis were performed offline.

**FIGURE 2:**
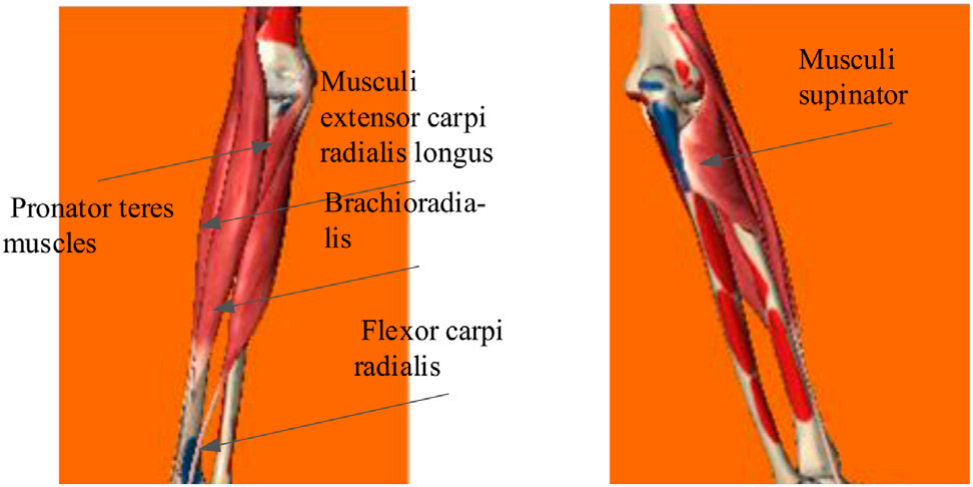
Selected muscle lateral freedom of the forearm movement.

The collected data needed preprocessing so as to extract features. The procedures included amplification, de-biasing, the bandpass filter, and the Kalman filter. Since the output signals contained 2.5 V bias voltages, it was necessary to subtract 2.5 V from the obtained signals to eliminate the effect. The filter method adopted was the finite impulse response digital bandpass filter. According to the studies (Baocheng Wang et al., 2014; Long et al., 2016), the scope was 20–200 Hz, and a notch filter was performed. The notch is set between 48 and 52 Hz to avoid the interference of the 50 Hz power frequency. The lower arm muscles are mainly composed of brachioradialis, flexor carpi ulnar, flexor carpi radialis, teres pronator, extensor carpi radialis longus, extensor carpi ulnar, polpolissimus brevis, pronator, extensor index finger, extensor digitus, and extensor digitus little. The brachioradialis muscle mainly flexes the elbow, rotates the forearm in and out, and maintains the median position during proximal fixation; the flexor carpi radialis is mainly used for flexion of the radial wrist joint, participating in wrist abduction, assisting elbow flexion and forearm internal rotation; the pronator teres muscle is mainly used to rotate the forearm inward to assist elbow flexion. Musculus extensor carpi radialis longus is mainly used to extend the wrist joint near fixation and participate in radiocarpal abduction and elbow extension and rotation; the pronator teres muscle is mainly involved in arm backrotation. According to the relationship between muscles and lateral movement, the electrode placement was pronator teres muscles, flexor carpi radialis, musculus extensor carpi radialis longus, musculus supinator, and brachioradialis, as shown in Figure 2.

### 2.2 Features

People could recognize the delay when the time is longer than 250 ms, so the data threshold of 250 ms was used as a set to get features. Besides, it was also critical to choose appropriate features to ensure classification accuracy. (Phinyomark et al., 2012) discussed the significance of selecting appropriate extraction feature methods. This study used mean absolute value (MAV), discrete Fourier transform (DFT), and wavelet transform (WT) as features (Yang et al., 2014).

(1) MAV

$$\mathrm{MAV}=\frac{1}{N}\sum_{n=1}^{N}\left|x\left(n\right)\right|$$ (1)

where *N* is the number of the sample, and *x*(*n*) are signals.

(2) DFT

The Fourier transform converts signals to the frequency domain. SEMG collected are discrete, so DFT is needed for *x*(*n*). The transformation formulas are defined as

$$\left\{ \begin{array}{l} X\left(k\right)=\sum_{n=0}^{N-1}x\left(n\right)W_{N}^{nk}\\ x\left(n\right)=\frac{1}{N}\sum_{k=0}^{N-1}X\left(k\right)W_{N}^{-nk} \end{array}\right.$$ (2)

where $W_{N}=e^{-j\frac{2\text{π}}{N}}$ and *N* is the number of samples.

(3) WT

For the signals *x*(*t*), the wavelet transformation is obtained as

$$W_{f}\left(a,b\right)=\frac{1}{\sqrt{a}}\int_{-\infty }^{\infty }x\left(t\right)\psi_{a,b}\left(\frac{t-b}{a}\right)dt$$ (3)

where $\frac{1}{\sqrt{a}}\psi_{a,b}\left(\frac{t-b}{a}\right)$ is the selected wavelet sequence, *a* is the scaling factor, *b* is the translation factor, and *a*, *b* ∈ R and *a*≠0.

### 2.3 The Continuous Motion Estimation Model

There was a close relationship between the sEMG and the joint angles during the execution of specific movements (Ding et al., 2017; Bergil et al., 2021; Makaram et al., 2021). Choosing the appropriate feature extraction method and machine learning algorithm was imperative for the human body motion angle estimation. Compared with KNN, LDA, and other processes (Liao et al., 2020), the BP neural network was appropriate to the case where the amount of classification data was small, and the classification results were extensive. Due to the limited training data in this study, a BP neural network model of sEMG features and motion angles was established to realize the human motion angles estimation. The model’s input signals were the features of sEMG, and the output signals were the movement angle of the upper limb.

**FIGURE 3:**
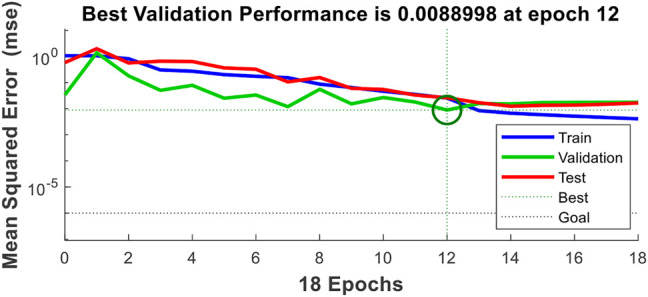
Result of the BP model.

The input layers of the BP estimation model contain fifteen neurons, the hidden layers six neurons, and output layers one neuron. The transfer functions of the hidden and output layers were tansig and purelin, respectively; the training number, training speed, and target error were 10,000, 0.01, and 0.001 (Chen et al., 2018). The training result of an individual is shown in Figure 3, from which we can see that the performance of the BP model is satisfying by using the corresponding parameters.

### 2.4 Kalman Filter

SEMG was utilized to predict the joint movement angle, but with its strong nonlinearity and the angle estimation of the pronation–supination movement controlled by many different muscles, the estimation results had a significant error. The nonlinear Kalman filter was employed to modify the estimation result.

The essence of the Kalman filter is to estimate the system’s operating statement based on the data obtained in the past and realize the estimation and correction functions. The discrete nonlinear control model is obtained as

$$x_{k}=f_{k-1}\left(x_{k-1},u_{k-1},w_{k-1}\right)$$ (4)

$$z_{k}=h_{h}\left(x_{k},v_{k}\right)$$ (5)

where *k* and *k*-1 are two consecutive moments, $\mu_{k-1}\in R^{r}$ is the input matrix of the system at time k-1, xk is the state of the system at time k, and $w_{k}\in R^{p}$ and $v_{k}\in R^{q}$ are Gaussian white noise, and there is no correlation between the two.

The statistical relationship between wk and vk is given as

$$\{\begin{array}{l} E\left(w_{k}\right)=q_{k},\mathrm{Cov}\left(w_{k},w_{j}\right)=Q_{k}\delta_{kj}\\ E\left(v_{k}\right)=r_{k},\mathrm{Cov}\left(v_{k},v_{j}\right)=R_{k}\delta_{\mathrm{kj}}\\ \mathrm{Cov}\left(w_{k},v_{j}\right)=0 \end{array}$$ (6)

where R*k* is a symmetric positive definite matrix, and ***Q*** *k* is a symmetric non-negative definite matrix.

The initial state *x*0 is independent of wk and vk. The mean and the covariance matrix of *x*0 are given by

$$\left\{ \begin{array}{l} {\hat{x}}_{0}=E\left(x_{0}\right)\\ P_{0}=\mathrm{Cov}\left(x_{0},x_{0}\right)=E\left[\left(x_{0}-{\hat{x}}_{0}\right){\left(x_{0}-{\hat{x}}_{0}\right)}^{T}\right] \end{array}\right.$$ (7)

The nonlinear control model f_k-1_ (·) is expanded into a Taylor series based on the filtered value ${\hat{x}}_{k-1}$ , and the second and higher orders are omitted, and xk is obtained as

$$x_{k}\approx f_{k-1}\left({\hat{x}}_{k-1},u_{k-1},q_{k-1}\right)+\frac{\partial f}{\partial {\hat{x}}_{k-1}}\left(x_{k-1}-{\hat{x}}_{k-1}\right)+\frac{\partial f}{\partial w_{k-1}}\left(w_{k-1}-q_{k-1}\right),$$ (8)

where fk-1 (·), xk-1, and wk-1 are given, respectively.

$$f_{k-1}\left(\cdot \right)={\left[f_{k-1}^{1}\left(\cdot \right)\;f_{k-1}^{2}\left(\cdot \right)\;\cdots \;f_{k-1}^{n}\left(\cdot \right)\right]}^{T};$$

$$x_{k-1}={\left[\begin{array}{cccc} x_{k-1}^{1} & x_{k-1}^{2} & \cdots & x_{k-1}^{n} \end{array}\right]}^{T};$$

$$w_{k-1}={\left[\begin{array}{cccc} w_{k-1}^{1} & w_{k-1}^{2} & \cdots & w_{k-1}^{p} \end{array}\right]}^{T};$$

$$\frac{\partial f}{\partial {\hat{x}}_{k-1}}={\frac{\partial f_{k-1}\left(x_{k-1},u_{k-1},w_{k-1}\right)}{\partial x_{k-1}}|}_{\begin{array}{c} x_{k-1}={\hat{x}}_{k-1}\\ w_{k-1}=q_{k-1} \end{array}}={\left[\begin{array}{cccc} \frac{\partial f_{k-1}^{1}\left(\cdot \right)}{\partial x_{k-1}^{1}} & \frac{\partial f_{k-1}^{1}\left(\cdot \right)}{\partial x_{k-1}^{2}} & \cdots & \frac{\partial f_{k-1}^{1}\left(\cdot \right)}{\partial x_{k-1}^{n}}\\ \frac{\partial f_{k-1}^{2}\left(\cdot \right)}{\partial x_{k-1}^{1}} & \frac{\partial f_{k-1}^{2}\left(\cdot \right)}{\partial x_{k-1}^{2}} & \cdots & \frac{\partial f_{k-1}^{2}\left(\cdot \right)}{\partial x_{k-1}^{n}}\\ \vdots & \vdots & & \vdots \\ \frac{\partial f_{k-1}^{n}\left(\cdot \right)}{\partial x_{k-1}^{1}} & \frac{\partial f_{k-1}^{n}\left(\cdot \right)}{\partial x_{k-1}^{2}} & \cdots & \frac{\partial f_{k-1}^{n}\left(\cdot \right)}{\partial x_{k-1}^{n}} \end{array}\right]}_{\begin{array}{c} x_{k-1}={\hat{x}}_{k-1}\\ w_{k-1}=q_{k-1} \end{array}};$$

$$\frac{\partial f}{\partial w_{k-1}}={\frac{\partial f_{k-1}\left(x_{k-1},u_{k-1},w_{k-1}\right)}{\partial w_{k-1}}|}_{\begin{array}{c} x_{k-1}={\hat{x}}_{k-1}\\ w_{k-1}=q_{k-1} \end{array}}={\left[\begin{array}{cccc} \frac{\partial f_{k-1}^{1}\left(\cdot \right)}{\partial w_{k-1}^{1}} & \frac{\partial f_{k-1}^{1}\left(\cdot \right)}{\partial w_{k-1}^{2}} & \cdots & \frac{\partial f_{k-1}^{1}\left(\cdot \right)}{\partial w_{k-1}^{p}}\\ \frac{\partial f_{k-1}^{2}\left(\cdot \right)}{\partial w_{k-1}^{1}} & \frac{\partial f_{k-1}^{2}\left(\cdot \right)}{\partial w_{k-1}^{2}} & \cdots & \frac{\partial f_{k-1}^{2}\left(\cdot \right)}{\partial w_{k-1}^{p}}\\ \vdots & \vdots & & \vdots \\ \frac{\partial f_{k-1}^{n}\left(\cdot \right)}{\partial w_{k-1}^{1}} & \frac{\partial f_{k-1}^{n}\left(\cdot \right)}{\partial w_{k-1}^{2}} & \cdots & \frac{\partial f_{k-1}^{n}\left(\cdot \right)}{\partial w_{k-1}^{p}} \end{array}\right]}_{\begin{array}{c} x_{k-1}={\hat{x}}_{k-1}\\ w_{k-1}=q_{k-1} \end{array}}.$$

Suppose $\frac{\partial f}{\partial {\hat{x}}_{k-1}}=\Phi_{k,\;k-1}$ , $\frac{\partial f}{\partial w_{k-1}}=\Gamma_{k,k-1}$ , and $f_{k-1}\left({\hat{x}}_{k-1},u_{k-1},q_{k-1}\right)-\frac{\partial f}{\partial {\hat{x}}_{k-1}}{\hat{x}}_{k-1}=U_{k-1}$ , then the first order linearization of the state function of the nonlinear model can be transformed into

$$x_{k}\approx \Phi_{k,k-1}x_{k-1}+U_{k-1}+\Gamma_{k,k-1}\left(w_{k-1}-q_{k-1}\right).$$ (9)

After expanding the nonlinear measurement function *h*_*k*_ (·) around the filter value ${\hat{x}}_{k|k-1}$ and omitting the Taylor series of the second and higher orders, the function *z*_*k*_ is explained as

$$z_{k}\approx h_{k}\left({\hat{x}}_{k|k-1},r_{k}\right)+\frac{\partial h}{\partial {\hat{x}}_{k|k-1}}\left(x_{k}-{\hat{x}}_{k|k-1}\right)+\frac{\partial h}{\partial v_{k}}\left(v_{k}-\gamma_{k}\right),$$ (10)

where *h*_*k*_ (·) and *v*_*k*_ are obtained by

$$h_{k}\left(\cdot \right)={\left[h_{k}^{1}\left(\cdot \right)\;h_{k}^{2}\left(\cdot \right)\;\cdots \;h_{k}^{m}\left(\cdot \right)\right]}^{T};$$

$$v_{k}={\left[\begin{array}{cccc} v_{k}^{1} & v_{k}^{2} & \cdots & v_{k}^{q} \end{array}\right]}^{T};$$

$$\frac{\partial h}{\partial {\hat{x}}_{k|k-1}}={\frac{\partial h_{k}\left(x_{k},v_{k}\right)}{\partial x_{k}}|}_{\begin{array}{c} x_{k}={\hat{x}}_{k|k-1}\\ v_{k}=r_{k\;\;\;\;\;\;} \end{array}}={\left[\begin{array}{cccc} \frac{\partial h_{k}^{1}\left(\cdot \right)}{\partial x_{k}^{1}} & \frac{\partial h_{k}^{1}\left(\cdot \right)}{\partial x_{k}^{2}} & \cdots & \frac{\partial h_{k}^{1}\left(\cdot \right)}{\partial x_{k}^{n}}\\ \frac{\partial h_{k}^{2}\left(\cdot \right)}{\partial x_{k}^{1}} & \frac{\partial h_{k}^{2}\left(\cdot \right)}{\partial x_{k}^{2}} & \cdots & \frac{\partial h_{k}^{2}\left(\cdot \right)}{\partial x_{k}^{n}}\\ \vdots & \vdots & & \vdots \\ \frac{\partial h_{k}^{m}\left(\cdot \right)}{\partial x_{k}^{1}} & \frac{\partial h_{k}^{m}\left(\cdot \right)}{\partial x_{k}^{2}} & \cdots & \frac{\partial h_{k}^{m}\left(\cdot \right)}{\partial x_{k}^{n}} \end{array}\right]}_{\begin{array}{c} x_{k}={\hat{x}}_{k|k-1}\\ v_{k}=r_{k}\;\;\;\;\;\; \end{array}}\frac{\partial h}{\partial v_{k}}={\frac{\partial h_{k}\left(x_{k},v_{k}\right)}{\partial v_{k}}|}_{\begin{array}{c} x_{k}={\hat{x}}_{k|k-1}\\ v_{k}=r_{k}\;\;\;\;\;\; \end{array}}={\left[\begin{array}{cccc} \frac{\partial h_{k}^{1}\left(\cdot \right)}{\partial v_{k}^{1}} & \frac{\partial h_{k}^{1}\left(\cdot \right)}{\partial v_{k}^{2}} & \cdots & \frac{\partial h_{k}^{1}\left(\cdot \right)}{\partial v_{k}^{q}}\\ \frac{\partial h_{k}^{2}\left(\cdot \right)}{\partial v_{k}^{1}} & \frac{\partial h_{k}^{2}\left(\cdot \right)}{\partial v_{k}^{2}} & \cdots & \frac{\partial h_{k}^{2}\left(\cdot \right)}{\partial v_{k}^{q}}\\ \vdots & \vdots & & \vdots \\ \frac{\partial h_{k}^{m}\left(\cdot \right)}{\partial v_{k}^{1}} & \frac{\partial h_{k}^{m}\left(\cdot \right)}{\partial v_{k}^{2}} & \cdots & \frac{\partial h_{k}^{m}\left(\cdot \right)}{\partial v_{k}^{q}} \end{array}\right]}_{\begin{array}{c} x_{k}={\hat{x}}_{k|k-1}\\ v_{k}=r_{k}\;\;\;\;\;\; \end{array}}.$$

Suppose $\frac{\partial h}{\partial {\hat{x}}_{k\text{|}k-1}}=H_{k}$ , $h_{k}\left({\hat{x}}_{k|k-1},r_{k}\right)-\frac{\partial h}{\partial {\hat{x}}_{k|k-1}}{\hat{x}}_{k|k-1}=y_{k}$ , and $\;\frac{\partial h}{\partial v_{k}}=\Lambda_{k}$ , then the first order linearization of the measurement function of the nonlinear system model is

$$z_{k}\approx H_{k}x_{k}+y_{k}+\Lambda_{k}\left(v_{k}-r_{k}\right).$$ (11)

The methods used to process the obtained angles are as follows: use eqs 9, 11 to convert the state model from nonlinear to linear, and then, use the basic linear equations of the discrete system for the Kalman filter.

### 2.5 The Range of the Angle

The exoskeleton robot of upper limbs has many DOFs, but it was not easy to foresee movements of all DOFs. The DOF of the pronation–supination movement was controlled by multiple muscles, so obtaining the most effective muscle combination was difficult. Moreover, the sEMG obtained was relatively weak and susceptible to external interference; thus, the angle estimation was complex. Existing studies, such as those by (Huang et al., 2020; Xi et al., 2021), are less involved in the DOF of the pronation–supination movement. This study took the DOF of the pronation–supination movement as the research object to expand the application range of the continuous movement estimation.

**FIGURE 4:**
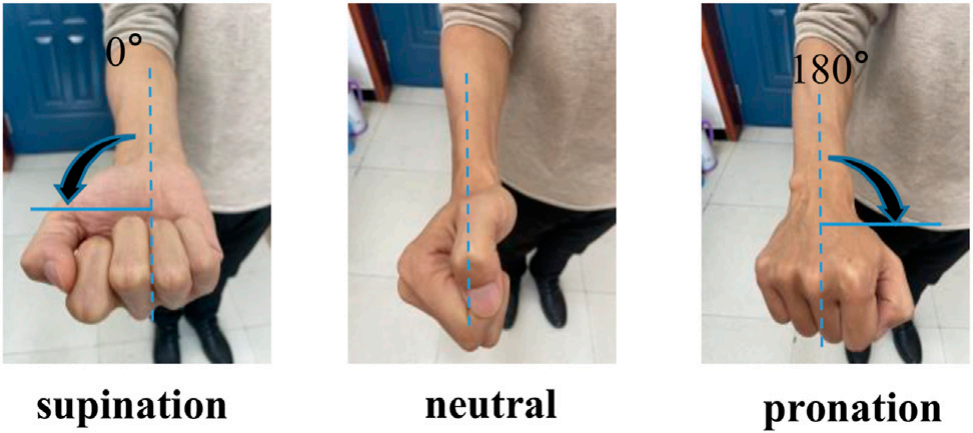
Pronation–supination movement angle.

An exoskeleton robot of the upper limb was used to verify the angle estimation accuracy and observe the error between the estimation and the human motion angle. Before the structure of the exoskeleton robot was defined, the human physiological structure was studied to determine the appropriate range of the DOF. The movement range could be gained according to ergonomic characteristics to ensure the safety of the exoskeleton robot during operation. The limit for the pronation–supination movement was -90–90.0°. At the same time, we defined that the center of the palm back to the ground was called 0° (see in Figure 4 supination), and the opposite was 180.0°. The movement begin the posion supination and ended the posiont pronation The angle definition of the DOF is illustrated in Figure 4.

The absolute error Δ and relative error *δ* of the joint rotation angle are respectively

$$\Delta =\left|x_{2}-x_{1}\right|$$ (12)

$$\delta =\frac{\left|x_{2}-x_{1}\right|}{x_{1}}\times 100\%$$ (13)

where *x*_1_ is the human body joint angle, and *x*_2_ is the exoskeleton robot angle. Two 9-axis attitude sensors (WT901C) are used to measure the *x*_1_ and *x*_2_.

### 2.6 Design Theory of One Degree of Freedom Exoskeleton Robot

To verify the effect of the continuous movement estimation on the exoskeleton robot control, we designed an exoskeleton robot with one DOF after a comprehensive consideration of the physiological structure of different upper limbs. In the designing process, we mainly considered the following key measures.

#### 2.6.1 Exoskeleton Robot Skeleton Design

The exoskeleton robot needed to be fixed on the subject’s upper limb and must be designed with a suitable clamping mechanism so that it could be fixed to the forearm. The overall structural design should be as light as possible and meet mechanical requirements. Aluminum alloy 6061 is a high-quality, low-density material produced by heat treatment and pre-stretching, which has several strengths: good processing performance with no deformation after processing, excellent anti-oxidation ability, and high toughness. Therefore, 6061 was chosen as the primary structural material. A porous structure was adopted without affecting the structural strength to make the overall structure as light as possible. The installation position of all motors was also carefully considered.

#### 2.6.2 Angle Sensor

The appropriate sensor should be chosen to measure the angle of one DOF. The exoskeleton robot’s pronation–supination movement angle was measured by a 9-axis attitude sensor (WT901C) with high accuracy and easy installation. The 9-axis attitude sensor was Shenzhen Weite Intelligent Company’s module, and the sampling frequency was 100 Hz, the sampling accuracy was 0.1°, and the sensor had the Kalman filter function. The data of 9 axes were three angular accelerations, three angular velocities, and three angles.

**FIGURE 5:**
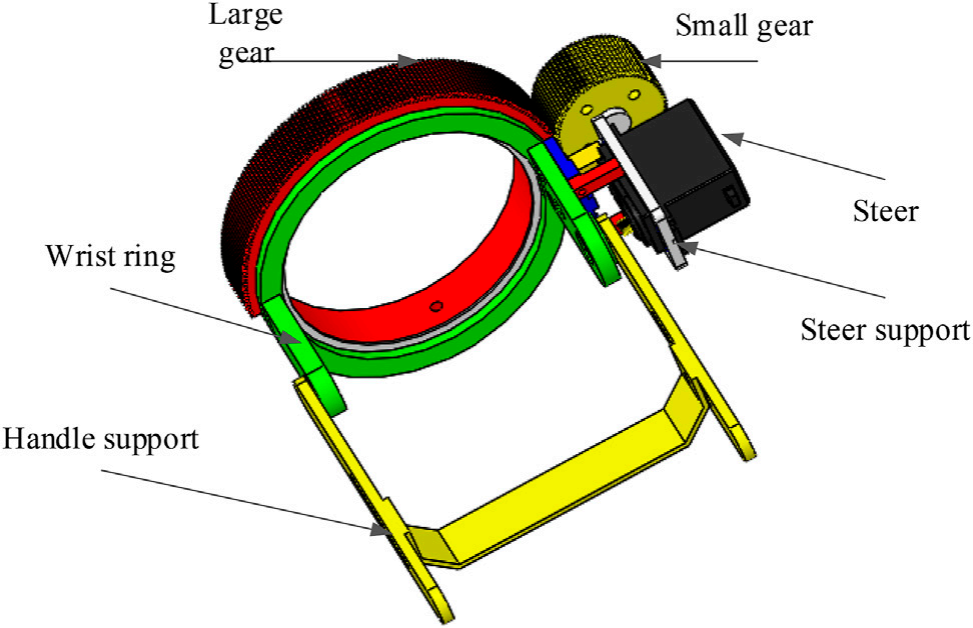
3D drawing of hand support structure.

**FIGURE 6:**
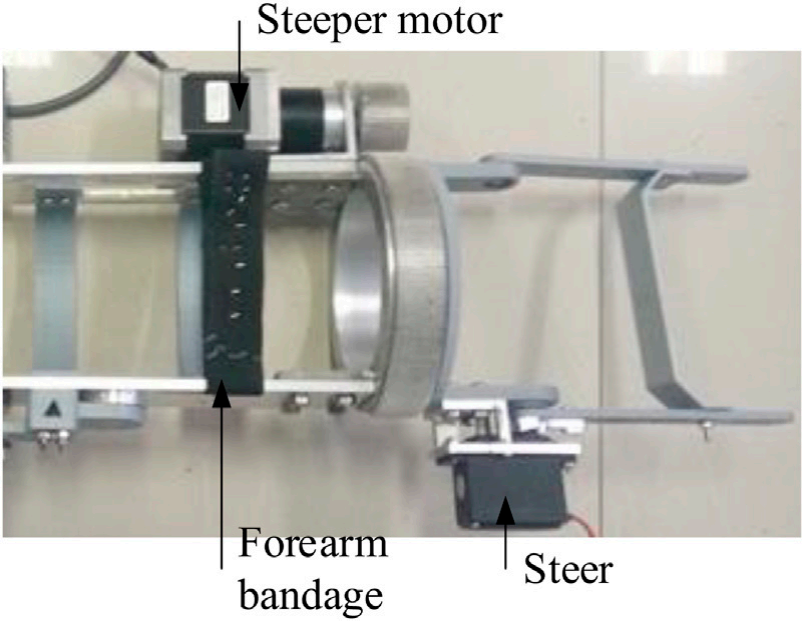
Physical model of the exoskeleton robot.

A three-dimensional model of the upper limb exoskeleton robot was designed considering the different size of subjects’ upper limbs (see Figure 5). The physical model of the one DOF upper limb exoskeleton robot was completed, as shown in Figure 6.

#### 2.6.3 Realization of the Exoskeleton Degree of Freedom

The process in which the exoskeleton drives the human forearm to realize pronation and supination DOF is as follows: the forearm is fixed to the transverse support structure (see Figure 6) through bandages, and the palm passes through the big gear hole and holds the handle support (see in Figure 5). The small gear drives the big gear to rotate and then realizes the forearm rotation.

### 2.7 Experimental Paradigm

One DOF was designed for the upper limb exoskeleton robot, which was then utilized to perform a tracking test. Then, a relationship model between sEMG features and the upper limb movement angle was set up. The participants in different trials vary from 5 to 20 according to the studies in (Pang et al., 2013; Geng et al., 2016; Wu and Chen 2021). The number of subjects in this trial was eight (including seven males, one female), aging between 23 and 32 years old, and six right-hands and two left-hands were included. Before attaching the EMG sensor, the electrode sticking site is kept clean and moist; an informed consent is signed; at least 2-min rest is needed between each group of movements in the trial to avoid the influence of muscle fatigue on the quality of sEMG. It was approved by the Medical and Experimental Animal Ethics Committee of Northwestern Polytechnical University.

The exoskeleton robot was fixed on the upper limb of a participant. The angle of the pronation–supination movement and the rotation angle of the exoskeleton robot’s wrist ring are measured by two attitude sensors. The attitude sensor lay flat on an individual’s palm, and the *x*-axis was collinear with the forearm (the individual was the subject). By analyzing the change, the pronation–supination movement angle could be obtained. Another attitude sensor was put on the wrist of the robot. The *x*-axis was collinear with the exoskeleton robot’s forearm, and the *x*-axis angle change was analyzed to obtain the rotation angle of the robot’s wrist ring. We could get lateral DOF’s tracking effect by comparing the differences between angles.

**FIGURE 7:**
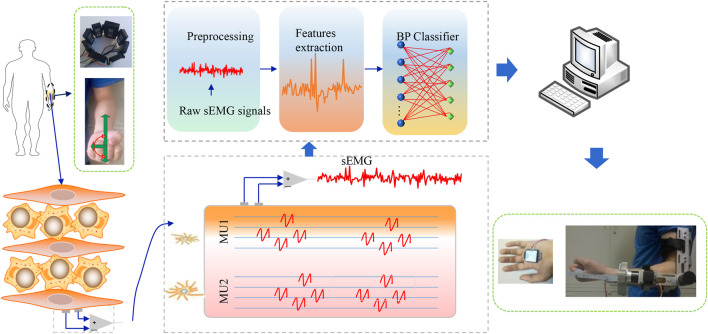
Experimental flow of controlling the exoskeleton robot.

During the test, the subject sat in a chair, keeping the body upright and looking straight ahead, and the angle between the upper arm and the forearm was 90.0°. Initially, the subject’s palm was parallel to the horizontal plane, and the palm was upward, and then rotated 180.0° until the palm was downward. The whole process lasted 5.0 s and tried to ensure a constant speed rotation, and other joints were kept as immobile as possible. During the participant’s forearm rotation with the exoskeleton robot, the DOF of the other joints should be kept as immobile as possible except for the lateral freedom of the movement. The subject’s forearm was rotated 180.0° laterally. The subject’s sEMG was employed as training data, and participants' signals were used as test data. Extracted features from the data and imported them into the BP neural network to generate an angle estimation model and used it to predict the lateral rotation angle of humans. Every subject received tests five times, and the results from five tests were averaged. The experimental flow is shown in Figure 7. First, the sEMG was obtained from the subjects and preprocessed. Then, the signals' feature was extracted, and finally, features were imported into BP for prediction. The predicted results are imported into the computer to drive the exoskeleton, and the tracking error is obtained according to the angle obtained from the exoskeleton and the actual rotation angle of the human body.

## 3 Results

**FIGURE 8:**
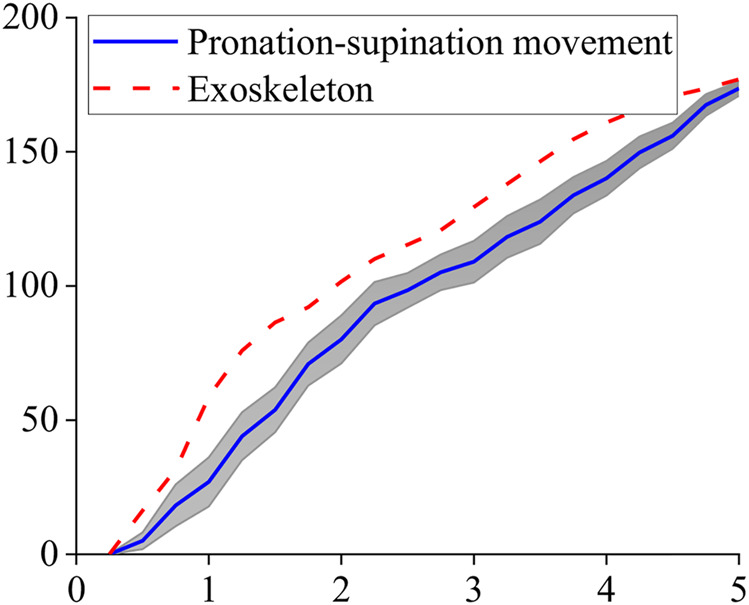
Tracking result of pronation–supination movement DOF.

**FIGURE 9:**
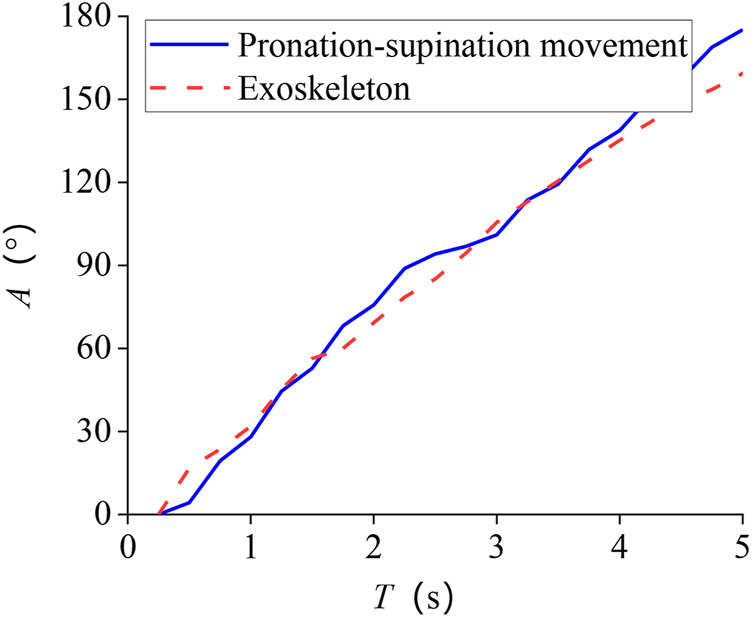
Subject 1 tracking results of pronation–supination movement DOF.

**FIGURE 10:**
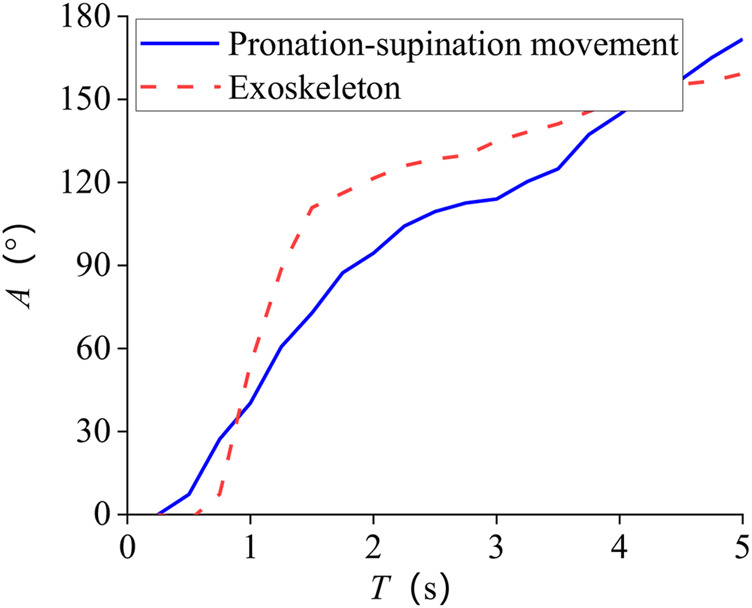
Subject 2 tracking results of pronation–supination movement DOF.

Tracking results of eight subjects’ mean are shown in Figure 8. The standard deviation of the whole process is 7.8°. One of the subjects’ tracking results are given in Figure 9 and Figure 10. The red dashed line represents the exoskeleton robot’s lateral angle; the solid blue line represents the rotation angle of the human body lateral movement obtained from the attitude sensor. The difference reflects the tracking error. It can be seen from Figures 8–10 that both the tracking effect of the average and the tracking effect of some individuals is accurate, and there is no significant fluctuation.

**FIGURE 11:**
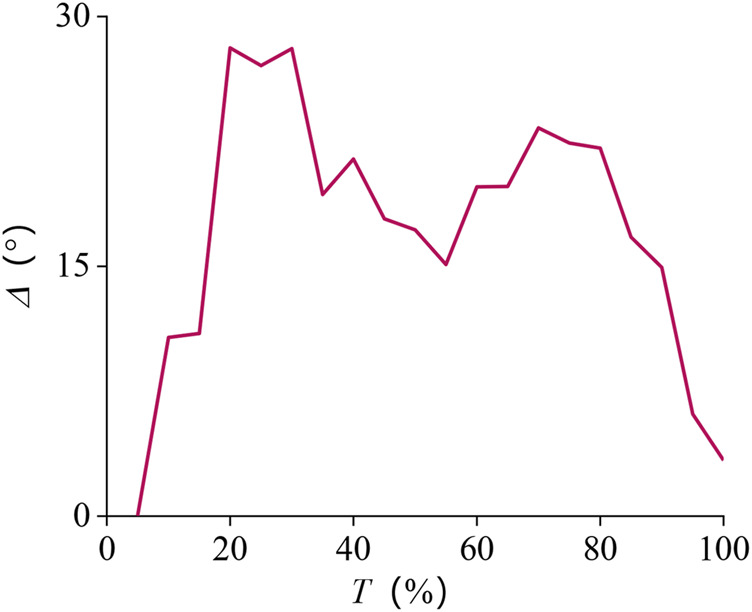
Absolute error curve of pronation–supination movement DOF.

We analyzed the error of the obtained tracking results using eq. 12, and the absolute error curve we obtained is shown in Figure 11. The movement time length was normalized between 0 and 100%. It can be seen that the error fluctuates around 15.0° during the entire period, and the average absolute error is about 17.6°.

Using eq. 13 to analyze the error results, to avoid the lateral forearm angle being too small, and causing a significant relative error, it took 1–5.0 s to explore it. The movement time length was normalized between 0 and 100%.

**FIGURE 12:**
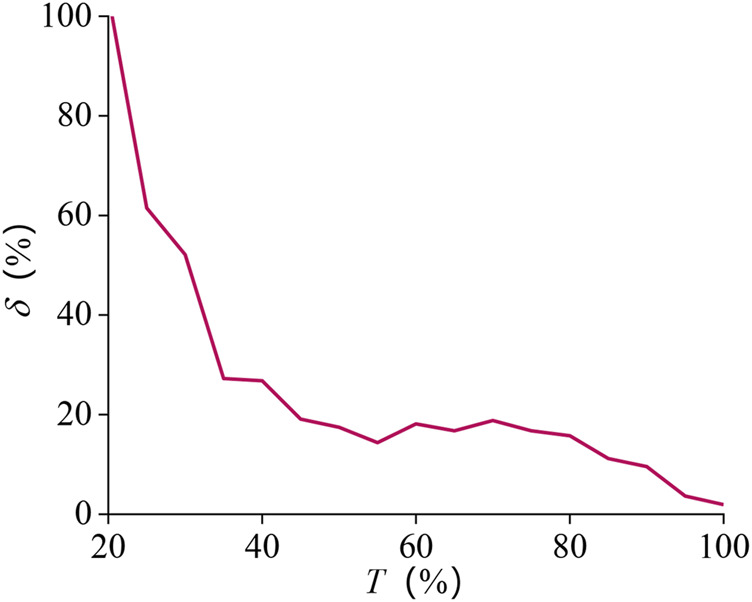
Relative error curve of pronation–supination movement DOF.

The results are presented in Figure 12. The error at the beginning is relatively large and finally stabilizes at about 15.0%, and the average value of the relative error is about 23.3%.

## 4 Discussion and Conclusion

### 4.1 Discussion

This study employed the neural network model as an alternative strategy to the Hill muscle model, which requires optimizing many different parameters. The designed BP estimation model could realize the forearm’s angle estimation: different subjects were selected to test the developed estimation model’s performance; then the average absolute and relative errors of results were obtained. The average relative error and the average absolute error was about 23.3% and 17.6°, respectively. The Kalman filter model is a good method to correct the predicted value. The results show that the proposed method also achieves good experimental results.

Scholars had conducted extensive research on the estimation of continuous movement of the human body. For example, (Han et al., 2015) selected the Hill muscle model to predict the continuous movement angle, which greatly improved the estimation accuracy of the upper limb motion. Jimson et al. (Vogel et al., 2013) proposed an activation model that parameterizes the electromechanical delay artificial neural network by extracting sEMG, estimating finger joints’ angle, and using the estimation results to drive the right-hand index finger exoskeleton robot to evaluate the effect. The Kalman filter estimates the current state based on the state of the last moment and estimates the optimal state through the correction of the estimated state and the observed state at the current moment, to obtain the optimal solution and realize the correction of the whole prediction process. Ang et al. (Pang et al., 2013) used the Kalman filter to process surface EMG signals and conducted experiments on five subjects. The results showed that the designed Hill muscle model could predict the angle of fingers when they naturally bent. In this study, the Kalman filter was used to modify the predicted value to improve the prediction accuracy of the pronation–supination movement DOF, and the BP neural network model was used to estimate the rotation angle of the human forearm, which expanded the freedom range of the upper limb continuous movement estimation, and improved the accuracy of the human–machine collaborative control process of the upper limb exoskeleton robot.

The rotation angle of the pronation–supination movement DOF was 0–180.0°. In future work, our estimation method needs to be improved to enhance accuracy, and the signals’ processing and analysis will be performed online. Figure 8 shows that despite the errors, the DOF is in line with the increasing trend of the actual angle, indicating that the errors do not affect the exoskeleton robot’s standard control.

## 5 Conclusion

This study presented the signals’ preprocessing and feature extraction, established the relationship model of sEMG and joint movement angle to realize the continuous movement estimation, and employed a designed exoskeleton robot to verify the model’s accuracy.

## Data Availability

The raw data supporting the conclusions of this article will be made available by the authors, without undue reservation.
